# Effectiveness of an integrated community and hospital digital health information system for maternal and newborn healthcare in Northern Kenya: a nonrandomized before-after evaluation

**DOI:** 10.3389/fdgth.2025.1584733

**Published:** 2025-12-17

**Authors:** Elizabeth Adhiambo Ombech, Hellen Gatakaa, Enock Oloo, Micah Oduol, Patrick Ben Ang'ela, Peter Etee, Gertrude Nasike, Wycliffe Khamala, Bonventure Ameyo, Sarah Lokaala, Samson Gwer, Moses Ndiritu

**Affiliations:** 1 Research and Evidence, Afya Research Africa, Nairobi, Kenya; 2 Health, Turkana County Government, Lodwar, Kenya; 3 Medical Physiology, Kenyatta University, Nairobi, Kenya

**Keywords:** antenatal care, skilled delivery, postnatal care, digital health information system, community health information systems, Turkana

## Abstract

**Background:**

Poor access to antenatal care (ANC), skilled delivery, and postnatal checks within 48 h of delivery are linked to adverse pregnancy outcomes. In Kenya, unequal use of these services has caused significant regional disparities, with 15 out of 47 counties being high priority.

**Objectives:**

To evaluate the effectiveness of a digital health solution to improve maternal and newborn health (MNH) uptake.

**Methods:**

From July 2017 to March 2019, we implemented an integrated community and hospital digital health information system, in ten health facilities and four community health units in Turkana County, Northern Kenya. We used a non-randomized before-after household survey. We assessed the proportion of mothers attending at least four antenatal visits, receiving skilled delivery, and receiving postnatal checks within 48 h at baseline and 12 months post-intervention. Statistical comparisons included *p*-values and 95% confidence intervals, accounting for clustering at the CHU level. These findings were compared with data from the Kenya Health Information System for the study subcounty and Turkana County.

**Results:**

Among a catchment population of 4,300 women of reproductive age (WRA), 692 and 608 women were interviewed at baseline and endline, respectively. STONE-HMIS® led to 5%, 23%, and 16% improvements in 4th antenatal care visits, skilled delivery, and postnatal checks within 48 h, respectively. For the same period, subcounty and county data showed that 57.7% and 65.8% of WRA attended at least 4 ANC visits, 39.5% and 67.8% delivered with skilled assistance, and 23.5% and 24% had postnatal checks.

**Conclusions:**

Integrating digital health systems at provider and community levels, aligned with health system priorities, showed marked improvements MNH indicators in an underserved, remote area.

## 1 Introduction

Turkana County in northern Kenya has one of the highest maternal mortality rates in the country. Similarly, approximately 1 mother dies for every 63 live births, and newborn survival is dismal (1). With a skilled delivery rate of 23.1%, access to optimal maternal and newborn health (MNH) services is limited (2). Geographical and logistical barriers complicate access in this expansive region. In addition, low levels of education, particularly among young mothers, impair optimal access to MNH care (3). Turkana mothers and their children are frequently mobile because of the realities of nomadic livelihoods. Mothers seek care, if available, from different healthcare providers in their path of movement. Thus, providers are unaware of complications previously identified by others, tracking the mother or patient at the community level is difficult, and patient education is ineffective. This is compounded by the fact that existing health information systems exist only at the facility level and are not linked to the community or across facilities. As a consequence, there is a significant loss to follow-up and inconsistency in MNH care, lowering the quality of care during pregnancy and the postnatal period. Clients are double registered across health facilities and community health units, resulting in grossly inaccurate aggregate data being collated into the DHIS2-based Kenya Health Information System (KHIS) (4).

Health systems need to be able to identify mothers across different providers, coordinate their care, and maintain quality in MNH care. Ideally, there should be collaboration between community health units and provider facilities to optimize service provision. Digital health information systems could help address the gaps in data systems if they are well designed, by informing better MNH public health strategies, rationalizing resource allocation, and optimizing individual clinical care. In sub-Saharan Africa, the use of digital technology in healthcare has been most prominent in the field of HIV/AIDS, where mobile phone texting and smart applications have been used to track adherence, pass information, send reminders and educate patients (5). As data complexity has increased and technology improved, disease-specific electronic medical records have become the norm. In MNH, digital systems have been used antenatal care (ANC) (6), electronic partographs during delivery (7), and childhood immunization (8, 9). However, these solutions are mostly uncoordinated and do not communicate with each other, leading to inefficiency and duplication of effort, particularly in low-resource settings (5). Importantly, a number of these digital health technologies have been developed far from the setting of their implementation and do not incorporate the necessary iterative feedback, thus becoming largely disconnected from the healthcare system (10).

These systems operate on the principle that harmonizing data across multiple points of care strengthens health system responsiveness and supports timely interventions throughout pregnancy, delivery, and the postnatal period (11–14).

To support access and coordinated MNH care in a setting with one of the worst MNH outcomes globally, we sought to implement an integrated digital health information system straddling community and health provider domains: the STONE-HMIS®. STONE-HMIS® is a modular web-based platform designed to uniquely identify mothers and newborns, synchronize community and facility data real time, track maternal and newborn health across the antenatal, intrapartum, and postnatal continuum, and interface with Kenya’s DHIS2-based national health information system. Developed in collaboration with end-users in Turkana, the system was co-created to align with nomadic population dynamics and existing workflows while ensuring interoperability with other digital health tools.

The design and implementation of STONE-HMIS® were informed by the Health Information Systems Interoperability Framework and the Technology Acceptance Model (TAM). Drawing from the interoperability framework, the system incorporated standardized data structures, biometric client identification, geospatial household mapping, and integration with DHIS2 to enable seamless, longitudinal tracking across community and facility levels. To facilitate adoption, key TAM constructs, including perceived usefulness and ease of use were embedded in system design and deployment. These included real-time defaulter tracing, minimal disruption to existing workflows, and continuous onsite user support (15).

The underlying hypothesis was that implementing STONE-HMIS® would improve skilled delivery rates, strengthen continuity of care, and enhance the accuracy and timeliness of MNH data reporting. This aligns with theoretical expectations that integrated digital systems, when interoperable and user-accepted, can reduce loss to follow-up, improve coordination, and lead to better clinical and public health outcomes (15). Understanding that digital health technologies augment and improve an existing health system, we approached the implementation as a complex intervention that has several components or interventions known to improve the experience of health workers, patient outcomes and the efficiency of a health system (16, 17). The aim of this study was to evaluate the changes in MNH indicators associated with the implementation of the STONE-HMIS®.

## 2 Materials and methods

### 2.1 Study objective and design

We evaluated whether STONE-HMIS®, an integrated community and provider modular health information system, had an effect on the proportion of expectant mothers who attended at least four antenatal care (ANC) visits, the proportion of women who delivered with a skilled birth attendant, and the proportion of newborn children who underwent postnatal checks within the first two days after delivery. This evaluation employed a nonrandomized before-after household survey design, chosen due to the programmatic nature of the intervention and ethical constraints around randomization in a high-need setting. A randomized design was not feasible because STONE-HMIS® was implemented as a health system–wide intervention in selected facilities and their linked communities. To mitigate potential biases inherent to the before-after design, baseline and end-line data were collected from comparable populations within the same community units, sampling procedures were standardized, and data collectors were trained uniformly.

### 2.2 Study setting

**Figure 1:**
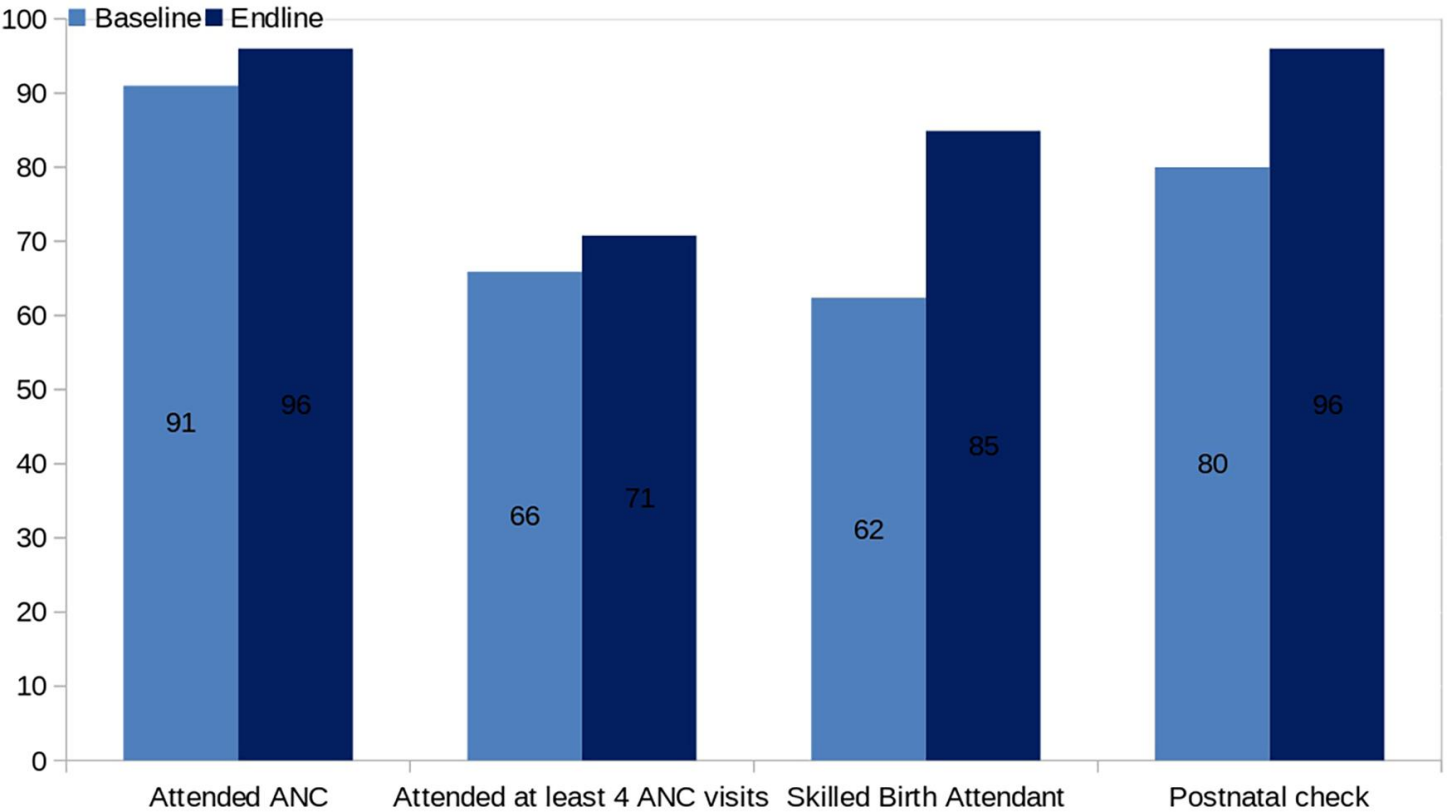
Bar chart comparing baseline and endline percentages for four healthcare metrics: Attended ANC (91 to 96), Attended at least 4 ANC visits (66 to 71), Skilled Birth Attendant (62 to 85), and Postnatal check (80 to 96). Dark blue bars represent endline values, light blue bars represent baseline values.

We implemented STONE-HMIS® in 10 select public and faith-based health facilities in the Loima and Turkana Central Subcounties (Figure 1). Within the Loima subcounty, community digital linkages were implemented in Lorugum, Kaitese, Nadapal, and Turkwel CHU. These four CHUs are affiliated with the St Elizabeth Mission and Lorgum Sub-County Hospitals, the Kaitese dispensary, the Nadapal dispensary, and the Turkwel health center, respectively. Due to the referrals and cross-utilization of hospitals in the adjacent Turkana Central subcounty, the St Patrick, St Monica, and St Catherine mission dispensaries, the Nagis dispensary, and the Lodwar County Referral Hospital were connected to track follow-up from the Loima subcounty. In Turkana Central subcounty, unlike in Loima subcounty, facility digital linkage was implemented without community health digital linkage. For the purpose of our evaluation, the Lorugum and Turkwel community health units (CHUs) in the Loima subcounty were conveniently sampled to participate in the baseline and end-line household surveys. These CHUs had a population of approximately 4,300 women of childbearing age. Loima subcounty is located in Kenya's remote northwestern region and is home to a nomadic pastoralist community known as the Ng'iturkana. Sixty percent (60%) of health care services are provided by faith-based and nongovernmental organisations. In 2014, skilled health personnel were responsible for 23% of deliveries in the subcounty (2).

### 2.3 Study population

The study target population was expectant mothers at any stage of pregnancy and mothers up to 12 months after delivery. Households were randomly selected using a simple random sampling approach from a comprehensive register maintained by community health workers (CHWs) in the linked health facilities. This register had been updated during the initial community mapping exercise for STONE-HMIS® implementation. Each household was assigned a unique identification number, and random numbers were generated using STATA to select the households for survey inclusion. Within the households, all mothers who were eligible, agreed to participate in the study and provided informed consent were interviewed by community health volunteers using a standard questionnaire. The refugee population was excluded because of international humanitarian norms restricting the record of the individual identity of people fleeing other countries to escape war, persecution, or natural disasters.

### 2.4 Study period

The project implementation period was 21 months, beginning in July 2017 and continuing through March 2019. Project start-up activities were undertaken from July 2017–January 2018. Baseline and end-line surveys were conducted in February 2018 and February 2019, respectively.

### 2.5 Study intervention and procedure

We implemented STONE-HMIS® in 5 provider facilities and their catchment communities in the Loima subcounty. Three of these facilities and their catchment CHU (Lorugum sub-County Hospital, St Elizabeth Lorugum Mission Hospital and Turkwell Health Centre) were the targets of our evaluation. The STONE-HMIS® was implemented across all the outpatient and inpatient MNH departments of these facilities. At the facility level, STONE-HMIS® serves all the components of the health system continuum, including administrative, clinical, pharmacy, and laboratory modules. It incorporates local and international databases such as county administrative units and dictionaries of medical terms (SNOMED and ICD). Health workers were trained on the use of the application, and trainers (TOTs) were identified to provide support. All patients presenting to the MNH departments of these facilities over the implementation period were registered and clerked using STONE-HMIS®.

Household data in the immediate catchment CHU were mapped into STONE-HMIS® in line with the Kenya Community Health Strategy. These data included household details (household heads, household relationships, selected household profiles and individual characteristics tracked by the Community Health Strategy), individual (finger) biometric identification of household members, and household GPS coordinates. A total of 1,780 households were registered in STONE-HMIS®. All expectant mothers were appropriately identified by community health workers (CHWs) during the initial mapping, and new mothers were subsequently identified through regular (weekly/monthly) visits across the households. Community Health Extension Workers (CHEWs) supervised every 10 CHWs and coordinated the follow-up of mothers for ANC, skilled delivery and postnatal care (PNC). Health workers worked with CHEW to follow up on MNH patients seen in their facilities, taking note of due visit dates for ANC, skilled delivery and PNC. Mothers who lapsed in their visit schedules were easily identified in STONE-HMIS® and actively followed up. At baseline and at the end of the survey, the interviewers visited households within the selected CHU and administered a standardized questionnaire to the eligible participants. The questionnaires sought details on ANC, skilled delivery and PNC attendance, verified where possible from the mother and baby ANC and PNC booklet (Additional Material 1: Study Questionnaire). Trained data collectors were identified from the local communities. The data collectors had a minimum qualification of four certificates and were fluent in the Kiswahili and local dialects. The training was performed for four days and covered data collection procedures, digital data collection methods, research ethics, confidentiality, autonomy, consent of participants and the voluntary nature of participation in the study. A pilot data collection exercise was performed to validate the tools and ensure that the data collectors were familiar with the tools. The trained language of the data collectors administered the questionnaire with support from the CHW. The questionnaire was administered using the Open Data Kit (ODK)^13^⁠ (18) tool, and the interviewee GPS details were tracked for authenticity. The questionnaire was tested prior to the assessment and revised to assure its validity and reliability. Regular spot checks were conducted by the project team to verify the authenticity of the collected data. When data were missing or had inexplicable inconsistencies, the gaps were resolved by referring to digital images where available and/or contacting the interviewers for clarification.

### 2.6 Data management and analysis

The study was designed to detect a 10% change in the proportion of women who deliver under a skilled birth attendant against a population prevalence of 30%, with a desired power of 80% and a 5% level of significance. Although this calculation focused on skilled birth attendance as the primary outcome, the sample size was adequate to detect changes of similar magnitude in secondary indicators, such as four or more ANC visits and postnatal checks, given comparable baseline prevalences and design effects. The formal sample size calculation was designed to detect a 10% change in skilled birth attendance from a baseline of 30%, with 80% power at the 5% significance level. *post-hoc* power estimates showed that, with the achieved sample size of 608 women at endline, the study also had approximately 80% power to detect a 10% absolute increase in ANC attendance from a baseline of 66% and in postnatal checks within 48 h from a baseline of 80%, assuming similar design effects. The estimated required number of respondents for each survey was 538 women of childbearing age. The data, excluding person identifiers, were stored on a central server with restricted access. The data were analyzed using Intercooled STATA version 13.0 (StataCorp 4905 Lakeway Drive College Station, TX 77845 USA)⁠ (19). Background characteristics (marital status, level of education, and source of income) were analyzed using Fisher's exact test to examine the hypothesis of independence between respondent characteristics at baseline and end-line. A *t* test was used to test the hypothesis of no difference in the mean age of the respondents between the baseline survey and the end-line survey. Descriptive statistics were used to summarize proportions of antenatal care, skilled delivery, and postnatal care at baseline and at the end of the study. Because sampling was conducted within Community Health Units (CHUs), observations within the same CHU were expected to be correlated. To account for this clustered design, all primary outcome analyses were performed using STATA survey (“svy”) commands with CHU specified as the primary sampling unit. This approach adjusts standard errors using Taylor linearization and provides cluster-robust 95% confidence intervals and *p*-values. For transparency, we present both the cluster-adjusted *p*-values from the survey analysis and the unadjusted *p*-values from standard chi-square tests, which do not account for clustering. This work follows a trajectory of implementation, study, and analysis similar to that of the work of a group in Western Kenya (20).

### 2.7 Data security, privacy and governance

STONE-HMIS® was designed and implemented using privacy-preserving principles consistent with international standards. A data-minimisation approach was applied throughout. Biometric identification relied on encrypted fingerprint minutiae templates, which were converted to non-reversible representations and linked to a numeric unique identifier used only within the system. No raw biometric images were stored. Household geolocation data were maintained solely by the local data controller and processor and were not transmitted to the central server, thereby reducing re-identification risk in line with the HIPAA Safe Harbor and Expert Determination frameworks. Only de-identified clinical and service-use data were synced to the central server, which was access-restricted and encrypted. Linkage across community and facility records required possession of locally stored keys or identifiers, ensuring that no single data store contained sufficient information for individual re-identification.

### 2.8 Ethical considerations

The study was approved by the AMREF Ethical Review Committee and was conducted in consideration of the ICH GCP guidelines on studies of human subjects. Written informed consent was obtained from the study participants during household visits. Confidentiality was maintained during the survey, and the study data were anonymised and maintained on servers accessible only by the principal investigators.

### 2.9 Limitations

This study used a before-after design without a control group, which limits causal inference and may be affected by secular trends or unmeasured confounding. Randomization was not possible due to the health system wide implementation approach and ethical considerations in withholding potential system benefits. To mitigate these limitations, we employed consistent sampling frames, comparable data collection methods at baseline and end-line, and statistical adjustments for clustering. Findings should be interpreted in light of these design constraints when generalizing to other settings.

## 3 Results

A total of 38,550 people were registered and clerked using STONE-HMIS® in the participating facilities: 10,502 were women of reproductive age, of whom 9,349 mothers attended ANC, skilled delivery and PNC clinics. A total of 1,780 households were mapped and registered in the community module. A total of 692 and 608 women of childbearing age were interviewed at baseline and at the end of the study, respectively.

The median ages of the respondents were 26 (IQR 21–31) and 25 (IQR 20–30) years at baseline and at the end of the study, respectively. The majority of the respondents were either unemployed, doing business or pastoralist; this was similar at baseline and at the end of the study. Most of the respondents were within a 5 km radius from the nearest health facility and had lived at their residence for more than one year. A summary of the other background characteristics is provided in Table 1.

**Table 1:** Characteristics of the respondents.

Characteristic	Baseline % (*n*)	End-line % (*n*)	*P* value
Mean age of respondents (in years)	64 (441)	67 (408)	0.788
Proportion by main source of livelihood:			
Unemployed	56 (387)	66 (401)	0.239
Business	24 (166)	8 (49)	
Pastoralist	11 (69)	20 (122)	
Farming/Agriculture	11 (69)	4 (24)	
Informal employment	0.3 (1)	1 (6)	
Formal employment	0.1 (0)	1 (6)	
Proportion by marital status:			
Currently married/living together	99 (685)	98 (596)	0.482
Single	0.5 (5)	1.3 (8)	
Separated/Divorced	0.2 (1)	0.2 (1)	
Widowed	0.3 (1)	0.3 (2)	
Proportion by highest level of education:			
No formal education	75 (591)	84 (511)	0.194
Primary school	9.5 (62)	11 (67)	
Secondary school	12 (83)	4 (24)	
College/University/Tertiary institution	4 (28)	1 (6)	
Distribution by distance of nearest health facility:			
Less than 5 km	67 (464)	84 (511)	0.118
6 to 10 km	8.8 (62)	15 (91)	
11 to 15 km	20 (138)	0.2 (1)	
16 to 20 km	2.3 (14)	0.5 (3)	
21 to 30 km	2.3 (14)	0.3 (1)	
31 to 40 km	0 (0)	0.3 (1)	
Distribution by duration of residence:			
Less than 3 months	21 (145)	23 (140)	0.129
4 to 6 months	13.3 (90)	9.4 (55)	
7 to 12 months	15 (104)	6.6 (43)	
More than 12 months	50.9 (353)	60.9 (371)	

The study area had greater coverage of antenatal care for at least 4 visits, skilled delivery and postnatal checks within 48 h of delivery compared to the average of the Loima subcounty, in which it is contained, and all of Turkana County, as shown in Table 2.

**Table 2:** Comparison of MNH indicators by locality.

Indicator	Coverage %		
	Study Area^a^	Loima Sub-County^b^	Turkana County^b^
	(*N* = 608)	(*N* = 31,596)	(*N* = 275,900)
ANC Attendance at least once	96	113.5	125.5
ANC Attendance at least 4 visits	71	57.7 (*p* < 0.01)	65.8 (*p* < 0.01)
Skilled birth Assistance	85	39.5 (*p* < 0.01)	67.8 (*p* < 0.01)
Postnatal check attendance	96	23.5 (*p* < 0.01)	24 (*p* < 0.01)

a Study area was a selection of Community Health Units from Loima Sub-County. The data are from the end-line survey.

b Average estimate from DHIS2 platform data for the period between the baseline and end-line surveys.

**Figure 2:**
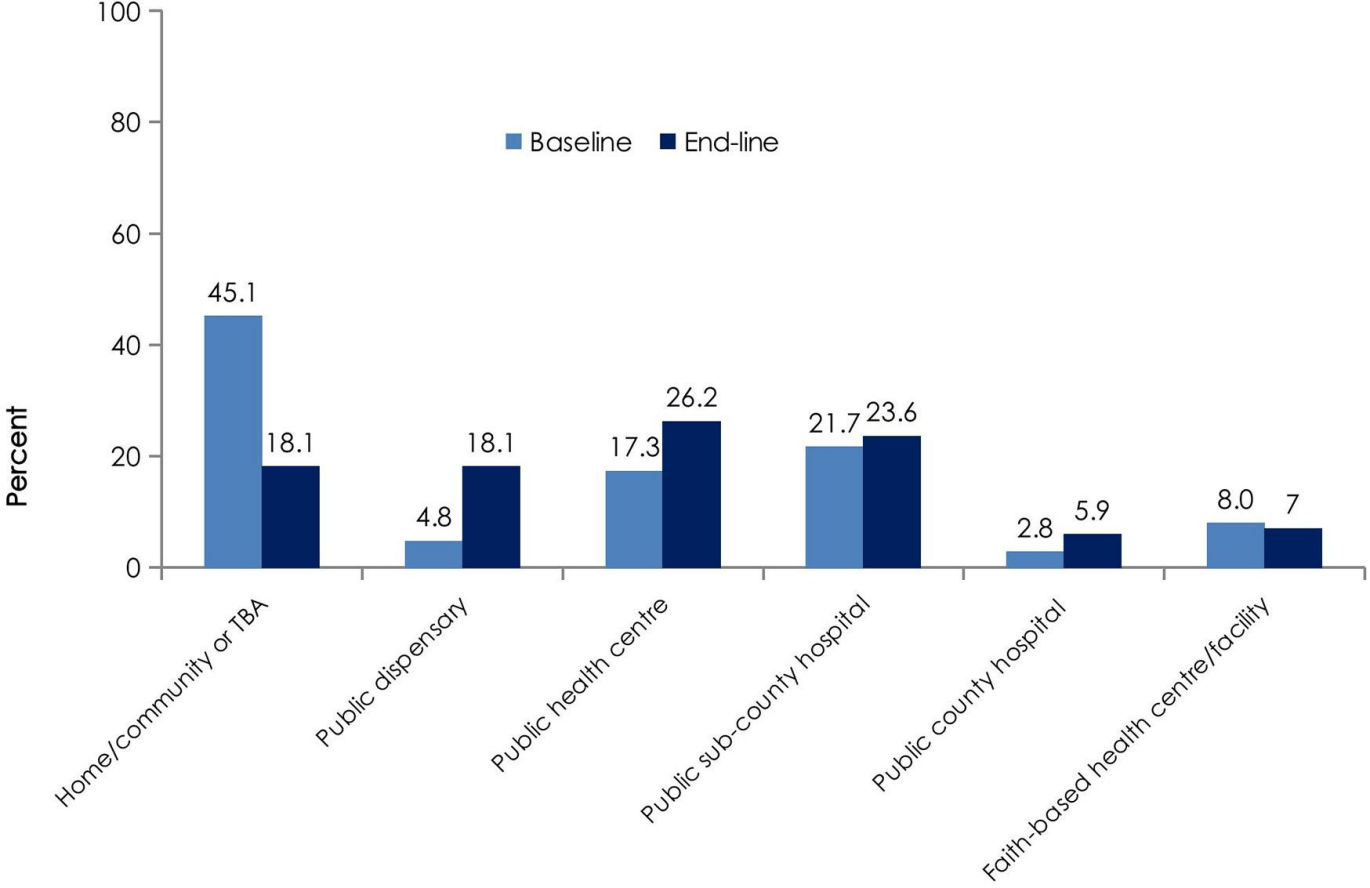
MNH indicators at baseline and end-line.

**Figure 3:**
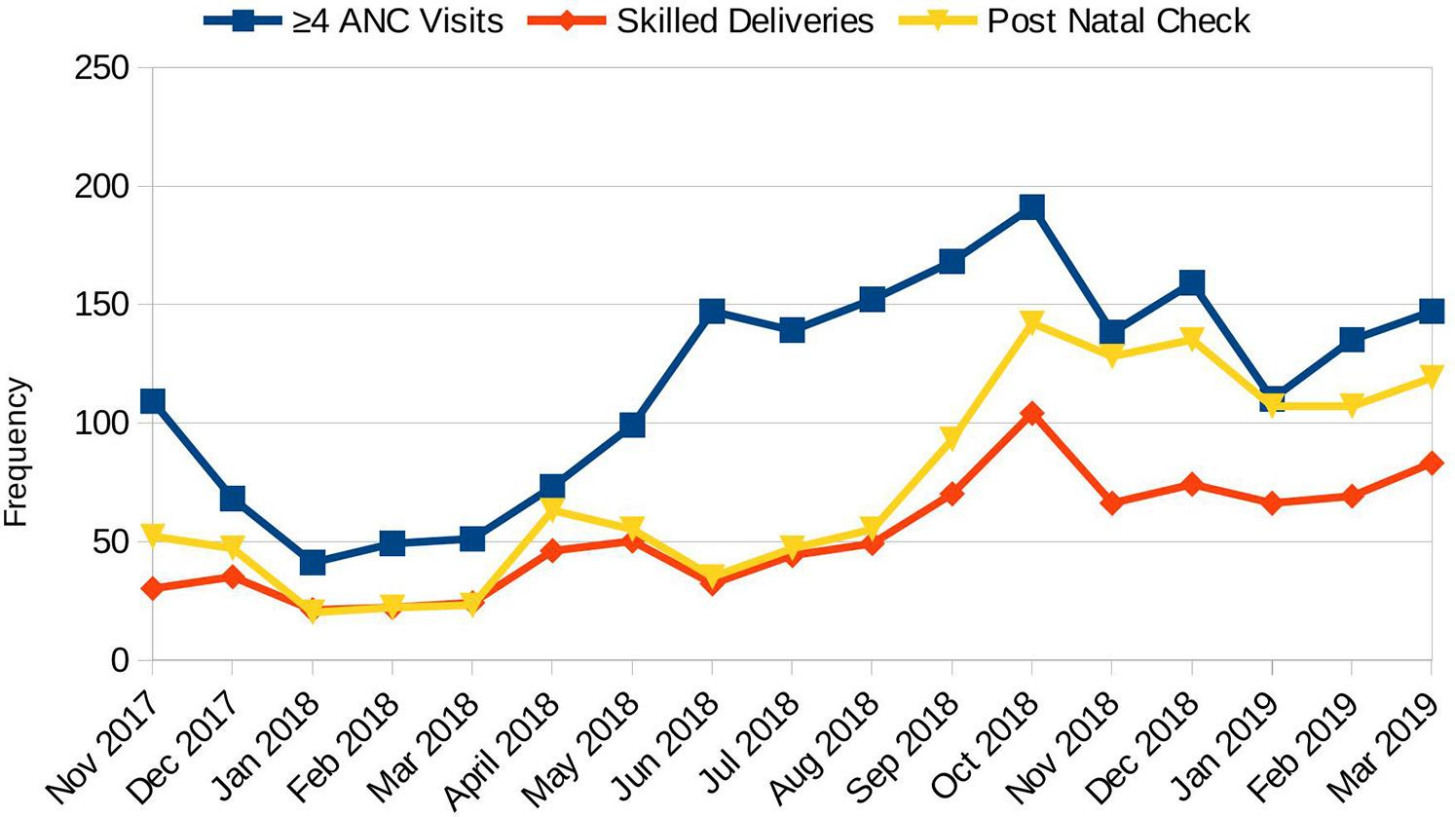
Monthly facility trends of MNH access. Monthly trends in access to maternal and newborn health services in intervention facilities, excluding the Lodwar County referral hospital.

Without accounting for clustering at the CHU level, there was a significant improvement in the MNH access indicators at the end-line compared to the baseline, as shown in Table 3. Figure 2 presents a comparative analysis of maternal and newborn health indicators at baseline and endline, demonstrating consistent improvements across all measured outcomes. The proportion of women attending at least four antenatal care visits increased from 66% to 71%, skilled birth attendance rose from 62% to 85%, and postnatal checks within 48 h improved from 80% to 96%. Figure 3 displays monthly facility-level trends in the utilization of MNH services. The number of women receiving at least four ANC visits, skilled deliveries, and postnatal checks increased progressively over the implementation period, with a steeper rise observed in skilled deliveries and postnatal checks.

**Table 3:** MNH indicators at baseline and end-line.

Indicators	Baseline % (95% CI)	*N*	End-line % [*n*] (95% CI)	*N*	*P* value	*P* value Chi-square test
Proportion of women who attended ANC during previous pregnancy	91 (61, 99)	503	96 (92, 98)	458	0.216	0.003
Proportion of women who attended four ANC visits during previous pregnancy	66 (59, 72)	458	71 (50, 85)	439	0.403	0.114
Proportion of women who delivered under skilled birth attendant	62 (32, 85)	503	85 (62, 95)	458	0.062	0.000
Proportion of women who delivered at the same health facility where they went for ANC	61 (25, 88)	503	82 (50, 95)	458	0.172	0.000
Proportion of mothers by timing of newborn examination after delivery:						
Within 1 day	68 (62, 74)	364	92 (49, 99)	421	0.122	0.000
Within 2 days	12 (8.8, 15.0)		4 (0.4, 29)			
After 3 days	6 (0.4, 53.7)		2 (1.9, 2.5)			
More than 2 days after delivery	13 (1.9, 56)		2 (<0, 61)			
Proportion of mothers examined after delivery of last child:	53 (42, 65)	503	84 (54, 96)	458	0.049	0.000

In Table 3, the column “*p* values” reports results adjusted for clustering at the CHU level using STATA survey commands. These adjusted values provide more conservative estimates that account for the sampling design. The second column, labelled “*P* value Chi-square test,” shows unadjusted significance values from standard chi-square tests, which do not account for clustering.

As shown in Table 2, the intervention area recorded higher coverage of all key MNH indicators compared to Loima subcounty and Turkana County. The proportion of women completing ≥4 ANC visits was 71% in the study area vs. 57.7% in Loima and 65.8% in Turkana; skilled delivery coverage was 85% compared to 39.5% and 67.8%, respectively; and postnatal check attendance was 96% vs. 23.5% and 24%. All differences were statistically significant (*p* < 0.01).

**Figure 4:**
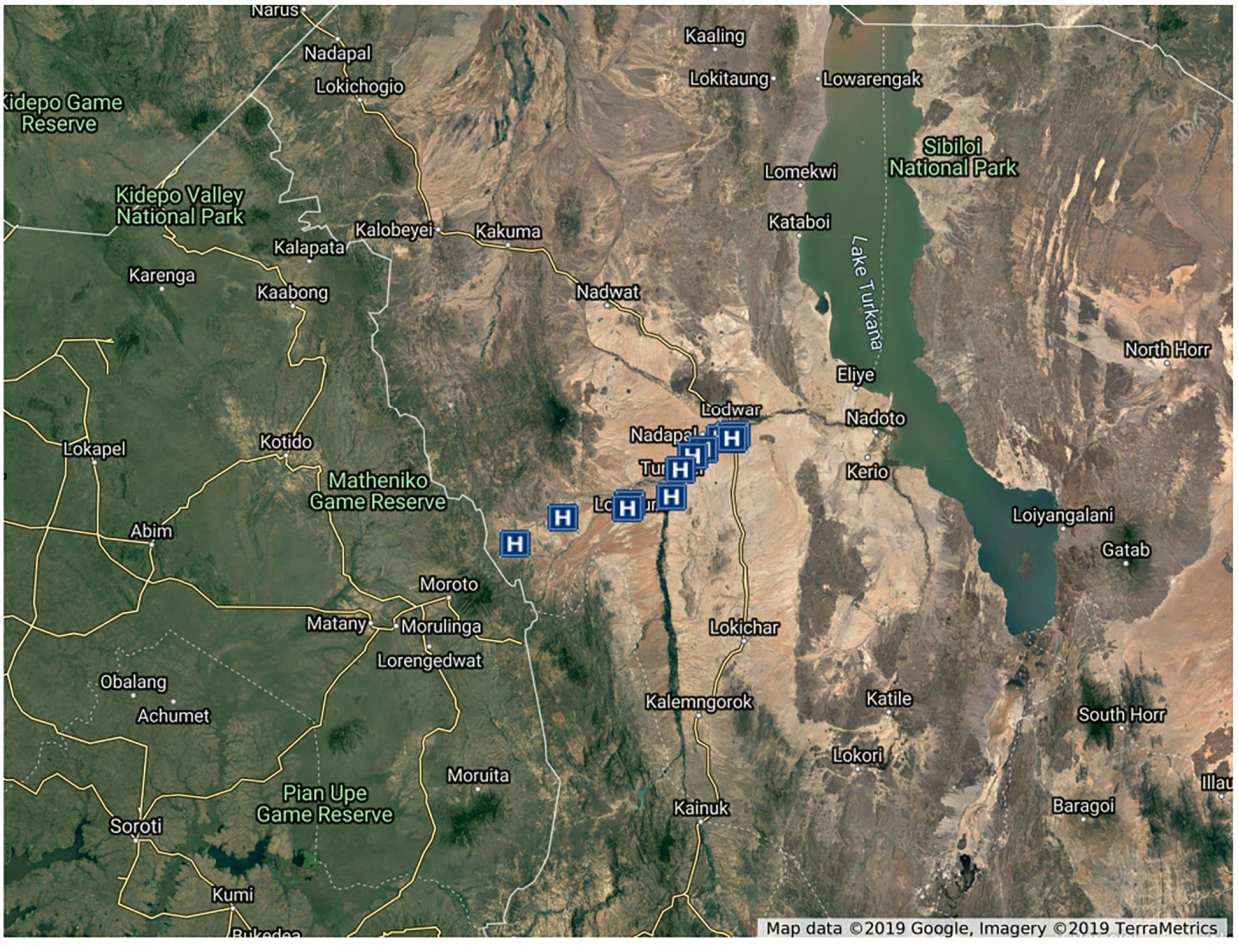
Satellite map of the participating facilities. Map of intervention health facilities in Turkana Central and Loima Constituencies of Turkana County, Kenya. Map from Google Earth, TerraMetrics, LLC.

Figure 4 shows the distribution of delivery locations at baseline and endline. The proportion of home or community-based deliveries decreased from 45.1% to 18.1%, while deliveries in public sub-county hospitals increased from 21.7% to 26.2%. Deliveries in public health centres and faith-based facilities also increased slightly over the same period. We also observed that fewer women (5%) experienced complications during pregnancy at the end of pregnancy than at baseline (16%).

## 4 Discussion

All the MNH indicators targeted by our project, including ANC attendance for at least 4 visits, delivery by a skilled birth assistant, and postnatal checks within 48 h of delivery, improved during the 12 months of the study. All maternal and newborn health indicators improved over the 12-month period. Attendance of at least four ANC visits improved by 5% (71% at endline vs. 66% at baseline, *p* = 0.114), skilled delivery improved by 23% (85% vs. 62%, *p* = 0.000), and postnatal checks within 48 h improved by 16% (96% vs. 84%, *p* = 0.000). After accounting for clustering at the CHU level, only skilled delivery and postnatal checks retained statistical significance. These findings indicate meaningful programmatic improvements, though not all met statistical thresholds. The absence of statistical significance for attendance of at least four ANC visits after adjustment for clustering may be attributable to the nature of ANC as a longitudinal service requiring multiple contacts over several months. Within the framework of a complex intervention such as STONE-HMIS®, changes in multi-visit services are influenced by numerous interacting components, including seasonal migration, competing livelihood demands, health worker availability, and facility access constraints. While the interoperability framework strengthened continuity of records and the TAM-informed design enhanced user adoption, the system defaulter tracing and follow-up functions are inherently more effective for discrete, time-bound services such as skilled delivery and postnatal checks than for services requiring sustained engagement across an extended period. In this context, it is plausible that skilled delivery and postnatal checks, as single-contact interventions, were more immediately responsive to the combined technical and behavioural components of the intervention, whereas ANC attendance remained more susceptible to external factors beyond the direct influence of the system. However, without accounting for clustering, statistically significant results were observed for skilled delivery attendance and newborn postnatal checks within 48 h of delivery. For the same period, aggregate data from the KHIS (21) for the County of Turkana and Loima Sub-County, where the study Community Health Units (CHU) are located, showed that all the MNH indicators under study except ANC attendance for at least one visit were significantly better in the CHU. The overestimation of average county- and subcountry-level ANC attendance at least once in the previous pregnancy indicates a fundamental problem associated with aggregate data, particularly in a highly migratory nomadic population where there is no unique personal identity or linkage of health data (22).

These results directly address our research objective of evaluating whether an integrated community and provider digital health information system could improve MNH service uptake. While improvements were observed in all indicators, the nonrandomized design limits our ability to attribute causality to the intervention.

The observed uptake and sustained use of the system by health workers aligns with constructs from the Technology Acceptance Model, particularly perceived usefulness and ease of use, both of which were reinforced through tailored training, real-time feedback, and integration into routine workflows. Similarly, the successful linkage of community and facility records reflects interoperability principles, including use of unique client identifiers, harmonized terminologies, and system alignment with DHIS2. Limitations encountered, such as incomplete identity documentation or infrastructure gaps, emphasize the challenges faced when applying these frameworks in resource-limited, nomadic settings.

As we have previously demonstrated with family planning data (4), information from KHIS and that from the Kenya Demographic Health Survey (KDHS) differ significantly due to the inaccuracy of the aggregate data. However, these results highlight the role of provider-pointing digital health technologies and systems as an integral component of the health system in increasing access to care for populations living in difficult-to-reach areas. The introduction of digital health systems has been observed to impact data quality (23), which could partly explain the observation. However, the twinning of digital health and programmatic interventions to improve goals of public health importance may be synergistic, as the effect was not observed in adjoining areas that were exposed to similar public health interventions but without digital health systems.

We developed a package of interventions supported by a modular electronic medical records system (Stone-HMIS) to create a digital health platform linking MNH service providers at health facilities and mothers and community health workers at community health units. We hypothesized that the education, awareness and tracking of mothers within their communities and as they interact with MNH providers in health facilities would increase access to MNH care. Furthermore, MNH service providers can access tools to enhance the quality of care they provide and assist in defaulter tracing. We approached this as a complex intervention that went beyond simple hardware and software introduction (16). The intervention had many interacting components: behavioral modification of health workers in learning and adopting digital health systems; the establishment and maintenance of digital infrastructure in a remote setting with rudimentary systems and extreme weather conditions; the use of biometric-based unique identifiers to facilitate data linkages across participating health facilities and the community; continuous adaptation to the needs and requirements of the health system as opposed to a set off-the-shelf digital system; and the provision of continuous technical support.

The linkage between the community health information system and provider facilities, which serve as resource centers for community health strategies, increased the bidirectional transmission and utilization of health information. This design overcame the observation that data tend to be collected for reporting purposes and not for decision-making at the point of collection (24). Often, information collected from community health units bypasses the resource centers, where the majority of the patients are attended, destined for the National Health Information System (KHIS). In addition, the digital information available from community health units is entirely aggregated and thus not amenable to direct use for patient care by linked resource health facilities. Aggregate data in national health information systems form the basis for planning and decision making. Studies show that aggregate data tend to be poorly utilized for system-level decision making in low-resource settings. Thus, its utility in directly improving the outcomes and quality of care provided to patients is subject to considerable debate internationally (25, 26).

Individual-level routine health service digital data were generated during patient care and management. The data were collated and aggregated automatically in line with the National Health Information System indicators and reviewed by record officers before transmission to the National Health Information System, although the capacity for direct machine-to-machine porting of the data existed. A numerical unique identifier based on an automated fingerprint identification system was issued to all patients during the lifetime of the project. This allowed for the tracking of individual clients and linkage of source documentation within the Ubuntu Digital Collaboration. To achieve privacy, deidentification was ensured through the HIPAA Expert Determination and Safe Harbor Rules (27). The integration of local administrative, pharmaceutical and health facility (KMHFL) databases and the use of standardized terminologies (SNOMED-CT & ICD 10) helped overcome the challenges of generating interoperable data in a setting where interoperability maturity for health systems is at a nascent level (28). This enabled the utility of both individual and aggregate level data in a remote setting, opening up the possibilities of using large amounts of data in changing patient experiences, outcomes and decision making at the point of care (26). Our attempt to utilize a foundational identity (national identification number) (22) for unique identification and health records linkage was precluded by the low numbers that possessed or carried national identification documents to health facilities. In addition, the national identity document is available to citizens only and issued to those 18 years old and older, which would leave out a large demographic of young mothers specifically targeted by the project.

We implemented the project with the knowledge that past use of digital health technologies, particularly mobile health applications, in similar settings was largely designed to meet the goals of disease- and program-specific surveillance and frequently resulted in a disarray of disintegrated systems (5). We encountered digital health systems/applications that were abandoned due to power and human resource problems—systems operating within the same physical location but on different networks and controls. We anticipated the risk of resistance to digital systems in health practice, which could lead to low acceptance, as well as frequent transfer of health workers due to service needs that caused attrition of staff trained and experienced in using digital health systems (29). To ensure adequate use of the digital health system, we built tools for monitoring specific work targeted by the project (MNH indicators) with routine health reporting, providing regular feedback combined with positive reinforcement in the form of extrinsic motivation and recognition with scheduled and on-demand retraining of health workers (30).

## 5 Limitations

This study has several limitations. The study utilized KHIS data to compare the rates of ANC attendance, skilled delivery and PNC visit attendance in the study area to subcounty and data where the intervention was not implemented. KHIS data may not reflect the true rates of ANC attendance due to double registration and incomplete records. In addition, the before-and-after study design can only imply an association but does not allow us to attribute a causal relationship between the intervention and the change in MNH indicators observed.

## 6 Conclusion

The implementation of an integrated provider facility and community-level digital health system and alignment with health system priorities and processes can facilitate the tracking and follow-up of expectant mothers and their linkage to care and seems to improve maternal and newborn health indicators in underserved and difficult-to-reach localities. Digital health systems should not be viewed as an end to themselves but as an integral part of the complex array of interventions that are synergistic to the achievement of health priorities and goals. This nonrandomized before-after study suggests that integrating community and facility digital health systems can improve maternal and newborn health indicators in underserved settings. However, causality cannot be inferred due to the study design and potential confounding factors. Future research should include randomized or longitudinal designs to confirm these findings and explore integration with national health information systems to enhance sustainability and scalability. Nevertheless, these findings illustrate how digital health interventions grounded in accepted interoperability and adoption frameworks can enhance maternal and newborn care in hard-to-reach settings, when adapted to local workflows and system priorities

## Data Availability

The raw data supporting the conclusions of this article will be made available by the authors, without undue reservation.
